# Supplementary material for: Voltage-dependent synaptic plasticity: Unsupervised probabilistic Hebbian plasticity rule based on neurons membrane potential
Source: Front Neurosci. 2022 Oct 21;16:983950. doi: 10.3389/fnins.2022.983950 (PMC9634260; doi:10.3389/fnins.2022.983950)
Supplement: Supplementary file 1 [file Data_Sheet_1.PDF]

Supplementary Material

Supplementary Material

1 Receptive fields for 50 output neurons

Increasing the number of neurons in the output layer makes multiple neurons learn different representations of each class.

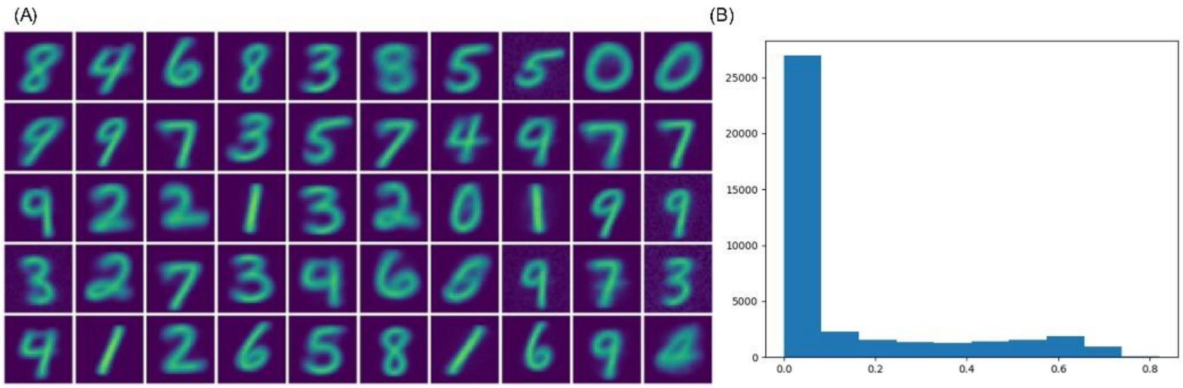

Supplementary Figure 1. A network of 784 input neurons and 50 output neurons was trained with 60,000 images from the training subset of the MNIST dataset over three epochs. The weight map from each of the 50 neurons is shown in (A) where each image represents 784 synaptic weights for each output neuron. The histogram of the synaptic weights is plotted in (B). A bimodal distribution can be observed.

2 Importance of weight dependence of weight update function

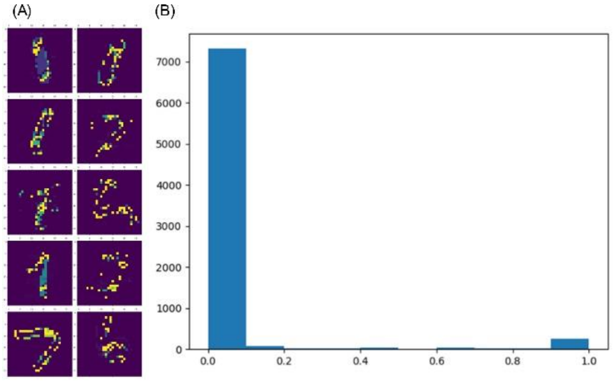

Supplementary Figure 2. Weight plots and histogram for additive VDSP. The change in weight (dW) is independent of the current weight (W). After training a network of 10 output neurons with 60,000 training images, the obtained weights for each of the output neuron is plotted in (A). The histogram of all the network weights is plotted in (B). It can be observed that the weights are set to either zero or one as in the histogram.

3 Importance of penalization of background pixels with bias

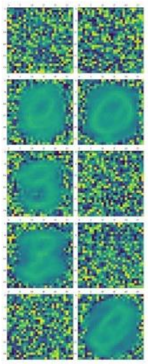

Supplementary Figure 3. Weights of the network when the bias of input neuron was set to zero. A bias of zero leads to membrane potential of input neurons representing background pixel to remain at zero. Hence, the weights of input neurons that were inactive were not depotentiated. The neuron to fire first after presentation of one image has a higher probability of firing even for other digits as some pixels overlap.

4 Sensitivity of the temporal VDSP window on LIF neuron's parameters

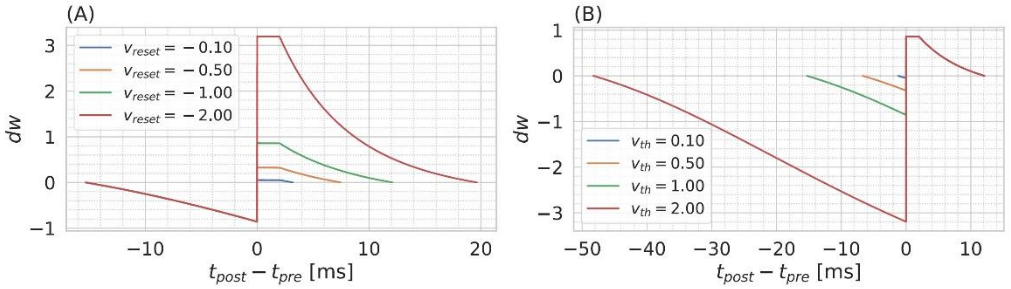

Supplementary Figure 4. Impact of the presynaptic LIF neuron's parameters on the shape of VDSP. In (A), the presynaptic neuron's reset potential is changed between -0.10 and -2, as indicated by the line colour with a fixed presynaptic neuron potential threshold of 1. This change impacts the potentiation part of the window ($tpost{-}tpre{>}0$). In (B), the presynaptic neuron's potential threshold is changed between 0.10 and 2.0 as indicated by the line colour, with a fixed presynaptic neuron reset value of -1. This change impacts the depression part of the window ($tpost{-}tpre{<}0$). Modifying these two values allows the tuning of the VDSP learning rule to a desired balance between potentiation and depression. I.e., for more potentiation, one should decrease the value of $vreset$ and for more depression, one should increase the value of vth.

5 Impact of additive gaussian noise on network performance for 50 output neurons

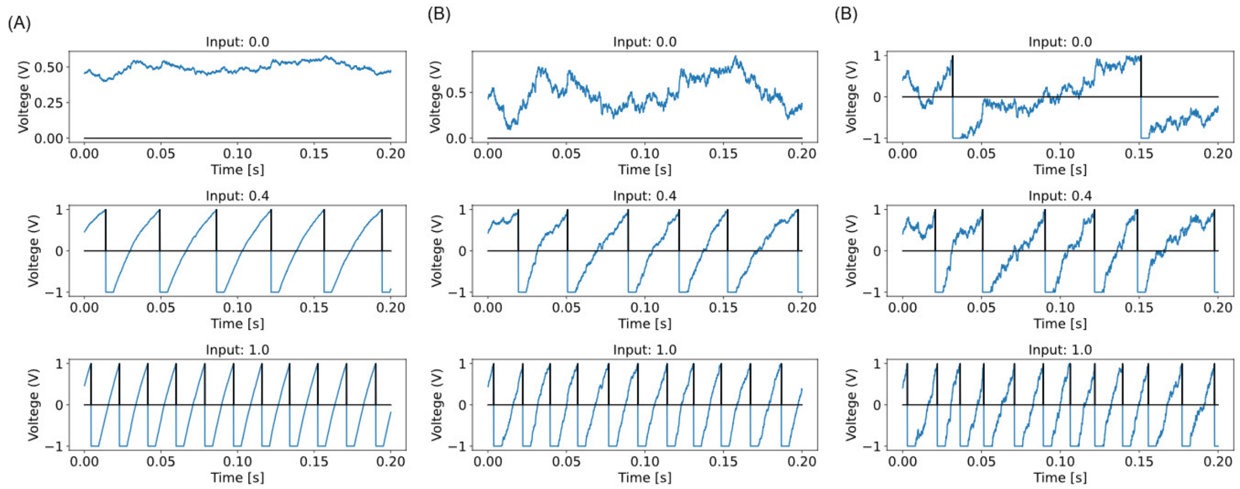

	Low noise	Mid noise	High noise
$Accuracy(\mu \pm \sigma)$	81.25 ± 0.65	80.75 ± 0.86	65.32 ± 1.31

Supplementary Figure 5. In (A), the neuron is excited by the constant input of magnitude 0, 0.4, and 1 to the input neuron of the MNIST classification network. Low magnitude noise of gaussian distribution centred around zero is injected to the input neuron in (A) for a network composed of 50 output neurons. In (B), the noise of mid-intensity is injected into the input neurons. Similarly, in (C), the noise of high intensity is injected into the input neurons. All accuracies are in format Mean ± S.D. resulting from five trials.

6 Impact of Poisson sampled input current on network performance for 10 output neurons

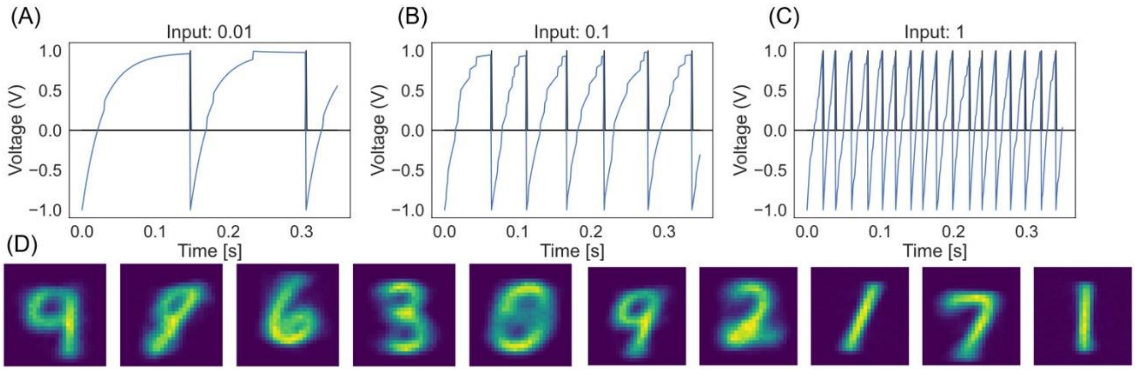

Supplementary Figure 6 In (A-C), a LIF neuron was stimulated by Poisson- sampled spike trains of 0.01, 0.1, and 1 kHz, with constant weight of 0.1 and bias b. (D) Poisson spikes with a frequency proportional to the pixel intensity of MNIST images and bias to penalize background pixels were fed to input neurons of SNN. The network with 10 output neurons was trained with 60,000 training images from the MNIST database with a maximum input spike frequency of 1kHz corresponding to a white pixel (intensity of 256). In (D), the weight of the individual output neuron is plotted to visualize the receptive fields at the end of training with Poisson spikes.
